# Exploring the barriers and facilitators to effective communication with people with age-related hearing loss in community pharmacy settings

**DOI:** 10.1016/j.rcsop.2025.100573

**Published:** 2025-05-13

**Authors:** Shanice Thomas, Jane Griffiths, Gabrielle Saunders, Denham Phipps, Chris Todd, Penny Lewis

**Affiliations:** a The University of Manchester Division of Pharmacy and Optometry, Manchester, United Kingdom; b The University of Manchester Division of Nursing Midwifery and Social Work, Manchester, United Kingdom; c The University of Manchester Division of Human Communication Development and Hearing, Manchester, United Kingdom; d National Institute for Health and Care Research (NIHR), Greater Manchester Patient Research Collaboration, Manchester, United Kingdom; e NIHR, Applied Research Collaboration Greater Manchester, Manchester, United Kingdom; f Health Innovation Manchester, Manchester, United Kingdom; g The University of Manchester Institute for Collaborative Research on Ageing, Manchester, United Kingdom; h Manchester University NHS Foundation Trust, Manchester, United Kingdom

**Keywords:** Community pharmacy, Older people, Hearing loss, Communication, Qualitative, United Kingdom

## Abstract

**Background:**

As populations age, there is a growing number of people who are affected by age-related hearing loss, who are living with chronic health conditions, treated using multiple medicines. Community pharmacy plays an important role in ensuring safe and effective medicine use.

**Objective:**

This study explored the barriers and facilitators to effective communication with people with age-related hearing loss in the community pharmacy setting.

**Methods:**

Semi-structured interviews were conducted with sixteen pharmacy users with self-reported age-related hearing loss in the United Kingdom (UK). Eight community pharmacists took part across two focus groups and one interview*.* Using a deductive-inductive approach to framework analysis, three overarching themes were generated.

**Results:**

‘Navigating the environment’ highlights barriers related to pharmacists reportedly high workloads and time pressures, also reflected in pharmacy user's accounts. Background noise reduced the confidentiality and effectiveness of communication. Participants had differing views on the extent to which hearing aids could overcome these challenges. ‘Debating the need to communicate and to disclose hearing loss’ reflects barriers relating to pharmacy users' tendency to not disclose their needs, in relation to their personal feelings (embarrassment), perceptions of, and limited contact with, community pharmacy services. Yet, pharmacists emphasised a need to know about hearing loss to adapt communication effectively. Participants reported similar and distinct perspectives regarding ‘coping strategies and solutions to communicate effectively’.

**Conclusion:**

Participants identified a need to improve pharmacists' capacity to implement communication adaptations for people with hearing loss, for which pharmacists suggested digital interventions, and to visibly recognise sensory needs, to promote disclosure.

## Highlights

• Pharmacists and pharmacy users saw a need for solutions to communication challenges.
• Environmental barriers limit the confidentiality and effectiveness of communication.
• Visibly recognising sensory needs in pharmacies could promote disclosure of need.
• There is a need to improve pharmacists' ability to implement communication strategies.

## 1 Introduction

Hearing loss is highly prevalent, affecting about nine million people in England, and this is projected to rise to one in five by 2035 due to population ageing.^1^ It is estimated to affect more than 40 % of people over 50 years old, and more than 70 % of people aged over 70 years.^1^ These figures are primarily driven by the onset of presbycusis: age-related hearing loss, which typically begins at around 50 years of age.^2^

Medication use is also prevalent in older people's lives: 80 % of people aged 65 to 74 years take at least one prescribed medication, with more than one in ten of those aged over 65 using eight or more prescribed medications per week.^3^ Older people are more likely to have multiple comorbidities and thus experience polypharmacy, a risk factor for adverse drug events.^4^ Therefore, ensuring older people can access and understand information about the medicines they are prescribed is crucial for their safe and effective use.

Hearing loss creates communication challenges, including in healthcare contexts.^5^^,^^6^ Inadequate communication is a commonly reported barrier to effective care for older people with hearing loss,^7^ and people with hearing loss are more likely to report challenges accessing healthcare than those without hearing difficulties.^8^^,^^9^ Specific to pharmacy, deaf people and those with age-related hearing loss have described a poor level of understanding of therapeutic regimens, dosages, and side effects related to their medications,^10^ which can lead to inappropriate medication use and preventable adverse drug events. Sign language users and people with age-related hearing loss have reported that they do not always hear complete medicine instructions; they may predict what the pharmacist has said,^11^ but give pharmacists the impression they have understood the instructions.^12^^,^^13^ During the COVID-19 pandemic, communication challenges for people with hearing loss were further exacerbated by the widespread use of face coverings and Perspex screens in healthcare settings.^14^

The Accessible Information Standard (AIS) mandates a consistent approach to identifying, recording, flagging, sharing, and meeting patients' communication and sensory needs in National Health Service (NHS) settings in England, UK.^15^ Despite it being mandated in law in England, researchers have raised concerns about the extent to which the AIS is applied in community pharmacy.^16^ Some community pharmacy staff have identified training needs^13^ and limited confidence when communicating with people with hearing loss.^17^

Whilst research has examined the perspectives of people with age-related sensory impairment, including hearing loss, and sign-language users in pharmacy settings, it is important to better understand the communication needs and experiences of people with age-related hearing loss when interacting with community pharmacists. This is particularly important as this form of hearing loss may be less evident and individuals are less likely to have developed coping strategies to manage the difficulties associated with newly acquired hearing loss. As inadequate communication could seriously impact the pharmaceutical care of this group, unpacking more specifically the factors that facilitate and impede effective communication between people with age-related hearing loss and pharmacists could help to identify possible solutions. This study therefore aimed to explore the perceived barriers and facilitators to effective communication with people with age-related hearing loss in the community pharmacy setting.

## 2 Method

### 2.1 Study design

This study involved semi-structured interviews with pharmacy users with age-related hearing loss; and focus groups and an interview with community pharmacists, to explore their subjective perceptions on the barriers and facilitators to effective communication. One-to-one interviews were conducted with pharmacy users, as online group discussions involving multiple voices could create noise interference and hearing difficulties, and to facilitate individualised communication adjustments as required. Practical reasons regarding the project timeline guided the decision to hold focus groups with pharmacists; interviews were offered for those who were unavailable for group discussions. Informed consent was obtained from each participant before each research activity. The study was ethically approved by the University of Manchester Research Ethics Committee (2022–13,524-22,018) and data were collected between March – June 2022. The Consolidated Criteria for Reporting Qualitative Studies (COREQ) guidelines was used in the reporting of this manuscript.^18^

### 2.2 Participants

Sixteen pharmacy users who self-identified as having hearing loss, aged 58 to 89 years, were interviewed. Of the eight pharmacists who participated, seven took part in one of two focus groups and one was interviewed. Pharmacists had an average of 20 years experience of professional practice, which ranged from 3 to 42 years. Three quarters of pharmacy users were male, and most were White British. Most pharmacy users used one or two hearing aids and self-reported that this improved their hearing abilities using a five-point scale (see Table 1). Pharmacy users had hearing loss for 13 years on average, though this ranged widely from 4 to 44 years.

**Table 1:** Demographic characteristics of pharmacy users with hearing loss (PU).

PU number	Gender	Age (years)	Ethnicity	Highest qualification	Number of hearing aids (HAs)	Hearing rating without (with) HAs	Years with hearing loss	Health rating	Number of chronic conditions
PU01	Male	63	White British	University degree or above	1	Poor (Good)	11	Good	0
PU02	Male	71	Asian Chinese	University degree or above	Eligible – refused	Fair (n/a)	5	Fair	2
PU03	Female	70	Prefer not to say	College	2, not used	Good (n/a)	4	Fair	1
PU04	Male	84	White	College	2	Poor (Good)	44	Excellent	1
PU05	Female	89	White English	University degree or above	2	Fair (Good)	5	Good	3
PU06	Male	85	White English	University degree or above	2	Poor–fair (Excellent)	15–20	Average	5
PU07	Female	58	White British	University degree or above	1	Poor (Good)	10	Good	2
PU08	Female	69	White British	University degree or above	2	Poor (Good)	15	Average	2
PU09	Male	79	White British	University degree or above	2	Fair–average (Good)	9	Good	3
PU10	Male	71	White British	College	2	Average (Average)	12	Average	2
PU11	Male	72	White British	College	0⁎	Fair (n/a)	10–15	Fair	2
PU12	Female	79	White British	College	2	Fair (average)	15	Excellent	3
PU13	Male	67	White British	High school	2	Poor (Good)	17	Good	1
PU14	Male	65	White British	University degree or above	0⁎	Good (n/a)	5	Poor	2
PU15	Male	71	White British	University degree or above	2	Fair (Good)	17	Good	1
PU16	Male	81	White British	University degree or above	2	Poor (Fair)	11	Good	1

⁎ Participants who have not been clinically assessed for their self-reported hearing loss.

### 2.3 Sampling and recruitment

Pharmacy users were recruited through the study team's personal and professional networks. This included a university bulletin; websites and social media pages hosted by the National Institute for Health and Care Research (NIHR) Applied Research Collaboration-Greater Manchester (ARC-GM), one of several regional collaborations between organisations funded by the NIHR that undertake applied health and care research, and Manchester Centre for Audiology and Deafness (ManCAD); and volunteer databases hosted by ManCAD and ‘Research for the Future’: a NIHR Clinical Research Network initiative. Pharmacists were recruited through the NIHR ARC-GM and the NIHR Greater Manchester Patient Safety Translational Research Centre, part of NIHR infrastructure; social media, mailing list adverts, professional networks, and snowball sampling.

### 2.4 Data collection

The study team developed the interview and focus group topic guides (see Supplementary Data) informed by a review of existing literature. [Author], a Research Assistant who had experience interviewing individuals with communication needs, led the data collection. All data collection activities with pharmacists and fourteen pharmacy users took place by video call. Two pharmacy users were interviewed by telephone. Prior to interviews, [Author] asked each pharmacy user about any adjustments they require to participate, including closed captions for online video calls. No participants opted for closed captions. Some participants stated they connected their hearing aid(s) to the digital device for the interview. One of the two pharmacy users who were interviewed by telephone asked [author] to slow down their speech at the beginning of the conversation. Based on the fact that responses to all questions seemed relevant and appropriate, hearing loss did not seem to disrupt communication during the telephone interviews for either user.

One focus group with pharmacists comprised five participants (CP01–05), while the other comprised two participants (CP06–07). Participants represented different pharmacy environments and had varying years of experience (see Table 2). Pharmacists were selected for a focus group depending on their availability. Whilst a mutually agreeable time for the focus groups was sought, this was not always possible. As such, an interview was conducted with one pharmacist (CP08) who was unable to join the focus group, so as to include their views in the study.

**Table 2:** Community pharmacists' (CP) professional characteristics.

Participant	Number of years in practice	Current community pharmacy environment
CP01	20	Independent
CP02	20	Retail/Large multiple provider
CP03	10	Retail/100-h pharmacy, based in General Practice
CP04	42	Independent
CP05	3	Retail/Large multiple provider
CP06	6	Retail/Large multiple provider
CP07	20	Distance selling pharmacy
CP08	22	Independent

[Author] collected pharmacy users' demographic characteristics and pharmacists' professional characteristics at the start of each discussion (Table 1, Table 2). This included pharmacy users' subjective ratings of their health status, hearing abilities with and without hearing aids, and number of chronic health conditions to provide context for their communication experiences. All interviews and focus groups were audio-recorded and transcribed verbatim by a university approved service or by [Author]. [Author] reviewed each transcript for accuracy and anonymised all personal identifiable information. Interviews with pharmacy users lasted for 47 min on average, ranging between 25 and 60 min. Both focus groups lasted about one hour, with the pharmacist interview lasting 35 min.

### 2.5 Analysis

The transcripts were analysed thematically, using the framework method^19^ to explore relationships and differences across the data and participant groups. [Author] undertook initial open coding of four transcripts, alongside two Masters students. This included three pharmacy user interview transcripts, and one pharmacist focus group transcript. Initial coding was carried out using a hybrid approach, with inductive coding to stay close to participants' expressed meanings and experiences around communication, and labelling relevant data under the deductive categories ‘barriers’ and ‘facilitators’ to effective communication. Recognising that communication is a two-way interactive process between pharmacy users and pharmacists, the study team sought to give full and equal level of attention to each participant, and participant groups' perspectives in the analysis, without privileging the viewpoints of one group. The whole research team met to agree on a set of codes to create an analytical framework, and to refine category definitions and assess the framework's validity and coherence. [Author] then input the framework into NVivo 12 software, and applied it to each transcript through a process of indexing,^19^ during which the framework was modified iteratively to reflect particularities within and between participant groups, and in relation to pharmacy users' demographic, and pharmacists' professional, characteristics. Indexed data were summarised into a coding matrix: a diagram of the similarities and differences across groups. The study team then mapped the matrix data into themes oriented around the ‘barriers’ and ‘facilitators’ to effective communication.

### 2.6 Trustworthiness

To ensure trustworthiness, the study aligned with guidance on multidisciplinary framework analysis,^19^ rigor in qualitative research,^20^ and COREQ to comprehensively report the findings.^18^ Credibility was ensured by [author] engaging in reflexivity,^21^ recognising the double outsiderness as a researcher without a clinical or pharmacy background, or lived experience of age-related hearing loss, though they have close family members with hearing loss. Reflexive practices included discussing interpretations in weekly multidisciplinary team meetings to interrogate assumptions about the data, to minimise bias as far as possible relating to disciplinary positionalities, which includes specialisms in clinical pharmacy services and patient safety [authors]; community nursing and long-term conditions [author]; auditory rehabilitation and audiology [author]; and healthy ageing [author]. [Author] has lived experience of hearing loss, and family members who do. Using semi-structured topic guides and documenting the data collection and analytic processes in detail, enabled a consistent approach to ensure the dependability of the findings. Confirmability was further supported through debriefing in weekly team meetings. Transferability was supported by reporting in detail the study sampling strategies, methodology, and participant contexts, so readers can assess how the findings apply to other settings, populations, and contexts.

## 3 Results

**Fig. 1:**
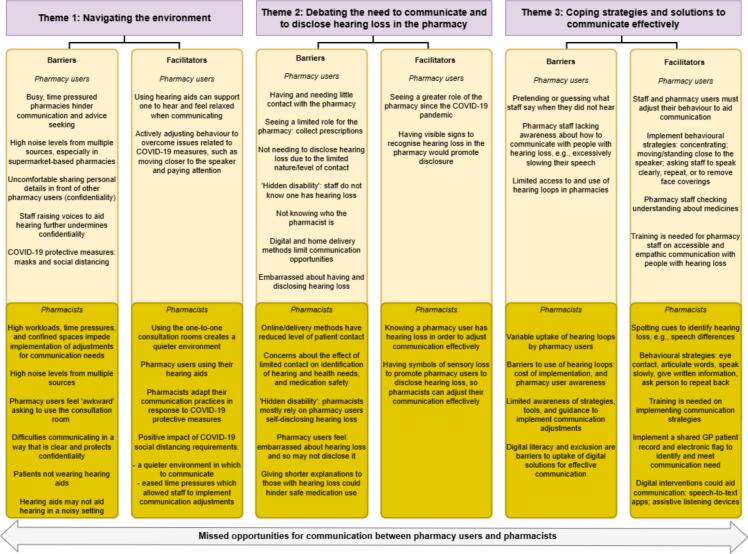
Thematic map of barriers and facilitators to effective communication from pharmacy user and pharmacist perspectives.

Factors that influence effective communication centred around three overarching themes: ‘*Navigating the environment’*; ‘*Debating the need to communicate and to disclose hearing loss in the pharmacy’*; and ‘*coping strategies and solutions to communicate effectively’*. Missed opportunities for communication was a cross-cutting thread running throughout participants' accounts. Relating to each theme, pharmacy users and pharmacists had distinct views on the barriers and facilitators to effective communication, as summarised in Fig. 1 and compared in the analysis below.

### 3.1 Navigating the environment

Both participant groups identified distinct environmental barriers and facilitators to effective communication, as detailed below.

### 3.2 Barriers

Pharmacy users consistently noted the ‘busy’ and time-pressured atmosphere in the pharmacy, particularly in relation to long queues for services, and staff appearing to be occupied with multiple tasks. As such, some felt rushed or that they could not take up time in the pharmacy, which created a barrier to seeking advice and limited communication to collecting prescriptions:

I wouldn't raise anything with them that was just on my mind and minor, simply because I'm aware of the pressure behind me [PU15].

Participants emphasised multi-layered barriers in the pharmacy environment, relating to high workloads, time pressures, and confined spaces which hindered their capacity to identify and meet the communication needs of people with hearing loss. Whilst pharmacists identified the private consultation room as offering a quieter environment for one-to-one communication, they did not always have time to implement this adjustment, or ‘take everyone to one side’ [CP01], due to intensive workloads. Pharmacists thus needed to communicate quickly and effectively:

There isn't always time to take someone to the consultation room, to sit them down in a quiet space and have a chat with… there isn't always space and time [CP07].

Both participant groups alluded to high levels of background noise, which can impact pharmacy users' ability to hear and thus communicate. For example, both groups identified noise from staff and customers talking, equipment, road traffic, and music. Pharmacists perceived noise as a major barrier inherent in the pharmacy environment:

The environment isn't naturally good for people with age-related hearing loss. You have a lot of background noise… staff having conversations in the background, endorsers, label printers [CP07].

Yet, pharmacy users had varying perspectives on the extent to which noise impacted their ability to hear and communicate, and which strategies were effective in addressing these challenges. Whilst creating difficulties for some, others found that noise either did not impede their ability to communicate effectively or they had “got used to it” [PU07]. Some pharmacy users regularly accessed pharmacies based in, sometimes along the aisles of, supermarkets, whilst others accessed these pharmacies as a ‘one-off’ when they struggled to obtain medications, in times of shortage. Supermarket pharmacies often had greater noise levels, especially from music, which impeded hearing and communication:

Sometimes it was seven or eight people in there, all waiting there for their prescriptions or talking amongst themselves, that was a little bit challenging [PU10].

Some pharmacy users stated they were uncomfortable when communicating personal details, such as their name and address, in front of other people. This was especially challenging when staff raised their voices to help them hear, which further reduced confidentiality:

I'm conscious of the fact though that they're quite often busy places community pharmacies, and it's very difficult to speak confidentially… Even the little booth… it wouldn't be an ideal place for discussing complex or particularly sensitive issues [PU01].

Pharmacists perceived that pharmacy users feel ‘awkward’ [CP05] or embarrassed about asking to use it. Reflecting pharmacy user accounts, some pharmacists noted problems with trying to communicate loudly enough so people with hearing loss can hear, whilst maintaining confidentially, in noisy and busy pharmacies:

If you're shouting someone's address to a patient to make sure they can hear… people who can't hear, they talk louder… if there's other people in the shop that's probably a barrier for them [CP08].

Pharmacy users widely discussed how the use of face coverings and plastic Perspex screens at counters, mandatory during the COVID-19 pandemic, exacerbated communication problems. Face coverings muffled speech, obscured facial expressions, and impeded comprehension related to lip reading, accentuating challenges for pharmacy users to hear and ‘respond accurately’ [PU16], thus acting as communication barriers. Some pharmacists explained how they adapted their communication practices when challenges arose relating to COVID-19 protective measures, to promote understanding about medications:

They can't even read our lips… handing out of medication over the pandemic that was definitely something that made me change my practice a bit because now I'll try and ask them about what medication is [CP06].

### 3.3 Facilitators

Pharmacy users and pharmacists had differing views on the environmental factors which facilitate effective communication. In particular, pharmacy users who wore hearing aids in the pharmacy said the aids helped them to hear and made them feel more relaxed about communicating, so they had ‘no problem’ with communication [PU05]. Whilst some pharmacists agreed that hearing aids could facilitate communication, many thought people often did not use hearing aids in the pharmacy, or that these aids would not be sufficient to overcome the high levels of noise, to support effective communication:

Background noise really affects their ability to hear things. Even if they're using hearing aids it will make a huge difference. They won't be able to hear as well [CP07].

Pharmacy users discussed strategies they used to overcome noise challenges, compounded by face coverings. For example, move closer to the speaker, pay close attention, and ask staff to speak clearly, repeat themselves, or remove face coverings. Pharmacy users identified a need for them to actively adjust their own behaviour in relation to such barriers, so they can hear and communicate effectively:

I'm sort of dealing face to face with the person you're speaking to… It may be busy in the surrounding, people talking and the background noise, but provided I'm near enough to the person I can hear what they're saying [PU01].

Whilst the COVID-19 pandemic created multiple communication barriers, some pharmacists suggested facilitative effects. For example, social distancing measures limited the number of people entering pharmacies at once, which created a quieter environment in which to communicate. This also made it ‘easier to manage customer flow’ [CP01], allowing more time to implement communication adjustments.

## 4 Debating the need to communicate and to disclose hearing loss in the pharmacy

Pharmacy users and pharmacists had differing views on the level and kind of communication needed within the pharmacy, and whether it is necessary to disclose hearing loss to facilitate communication in this context.

### 4.1 Barriers

Pharmacists emphasised the importance of communicating with people regarding their medicines. Despite this, many pharmacy users commented that they had and needed very limited contact and communication with pharmacists. This related to how they perceived the role of, and used, community pharmacy services, as a place to collect medications, not necessarily as a place to obtain health information or other services. As interactions with the pharmacy often only involved stating one's name and address to collect prescriptions, pharmacy users said they needed little communication with the pharmacy:

There's no issue because I don't ask any questions other than ‘I've come for my medication’. I don't have any relationship with them… I just want to get my prescription [PU03].

Most pharmacy users obtained their medications from one pharmacy. The relationship they had, or wanted, with that pharmacy varied, and some were not aware of ever speaking to the pharmacist, or they found it was “hard to know who the pharmacist is” [PU10]. Some pharmacy users' awareness about the wider role of pharmacy services emerged during the COVID-19 pandemic when they, or others they knew, were directed to the pharmacy by other healthcare providers as an alternative source of support:

Up until recently, just dispensing medicines. But obviously times are changing… pharmacists are being given more responsibilities… it's another avenue for the general public to get some help, because seeing a GP is becoming harder [PU11].

Most pharmacy users opted for digital prescription ordering, and some received home deliveries, because it was easy and convenient, saved travel time, offered better tracking of medications and a way of communicating with GP by adding notes to a repeat prescription request form. These methods of ordering and collecting prescriptions further reduced the level of contact pharmacy users had with their pharmacist:

There's very little communication needed. I put it through the app, I get a text to tell me when it's ready to collect, I go in and say my name and my address… and off I go [PU13].

Pharmacy users alluded to hearing loss as a ‘hidden disability’: without obvious, visible indicators, especially if hearing aids were not visible, worn, or purposely hidden. Many therefore suspected that staff were not aware of their hearing loss. As pharmacy users generally had predictable interactions in the pharmacy, providing only basic information about their name and address, few felt the need to disclose their hearing loss:

Not necessary… with the way that we order our medications, and we can check whether they've been approved within the surgery, then generally we know what we're going for. So, we don't have to go and ask to go into the private interview room [PU09].

However, pharmacists emphasised needing to know about, and have regular contact with, people who have hearing loss, to communicate with them effectively about medication use. Mirroring pharmacy users' accounts, pharmacists noted that many pharmacies saw increased adoption of online and/or delivery methods for obtaining medications since the COVID-19 pandemic. Whilst pharmacy users emphasised benefits, pharmacists had concerns about the reduced level of patient contact this involves; that people may not be managing their medications correctly, and that declining health and hearing may go unnoticed and untreated:

If patients are getting their medications delivered, there's less chance of communication… you sometimes wonder what's happening to those patients, how they're dealing with their medications, are they understanding everything? [CP02].

Like pharmacy users, pharmacists alluded to the lack of visible, physical indicators of hearing loss, making it difficult for pharmacists to know when someone has hearing loss. Pharmacists relied on people “self-identifying” hearing loss [CP03], to enable them to effectively adapt their communication. They suspected that pharmacy users ‘pretend they've understood’ [CP02] rather than disclose their hearing loss due to feeling embarrassed, especially when other people were waiting in the pharmacy.

Yet, pharmacy users had personal differences regarding how willing, able, and comfortable they were with disclosing their hearing loss, which seemed to relate to generational and gendered expectations. For instance, younger females described concealing their hearing loss by wearing their hair longer to cover hearing aids, or they conveyed embarrassment about having to repeatedly disclose hearing loss. Whereas others, typically male, older pharmacy users, had “no problem” with telling people to “speak up, I can't hear you” [PU04]:

It's a bit embarrassing having to keep saying every time… that just reinforces to me that I have a problem [PU07].

Whilst pharmacists emphasised a need for regular contact with pharmacy users regarding medication use, they found it difficult trying to implement communication adjustments, given workload and time challenges. In attempt to make verbal information as clear as possible, some pharmacists would “condense” their explanations about medications [CP03]. There seemed to be a fine line between providing a clear and concise explanation about medications, by explaining things “in as short a possible way, by using the least number of words” [CP04], and giving people enough information for safe medication use. Pharmacists with a longer history of experience in practice tended to, and may thus be more willing, able, and comfortable to, exercise these communication practices with people with hearing loss:

You do sometimes find yourself just shortening what you'd say down, which isn't really ideal. I don't think the idea that we should be giving people with hearing impairment less information is a particularly good idea [CP01].

### 4.2 Facilitators

Pharmacy users and pharmacists suggested similar ideas to promote recognition and disclosure of hearing loss in the pharmacy, to improve communication. Pharmacy users felt more should be done to acknowledge hidden disabilities, such as through visible signage, and implementing systems to keep records of hearing and communication needs, particularly to avoid the embarrassment that comes from repeatedly disclosing hearing loss:

I don't see any behavioural things or visual things in the pharmacy to say we are aware of or can help you in the following circumstances [PU09].

If they did have something like on your prescription or something that alerted them to that, so that the onus isn't always on me… to keep informing and keep saying that you're deaf… [PU07].

Pharmacists similarly highlighted the need for stronger ‘symbols’ relating to sensory loss, in order to promote disclosure and, thus, facilitate appropriate communication adjustments:

It's quite prudent to have some signs on either side of the counter that… if you need the help of a pharmacist, please ask… to create a symbol and then having that symbol as identified that we are here [CP04].

## 5 Coping strategies and solutions to communicate effectively

Whilst many pharmacy users did not disclose hearing loss in the pharmacy, and pharmacists perceived that people may not do so due to feeling embarrassed, both groups noted distinct coping strategies, and suggested solutions, to communicate more effectively.

### 5.1 Barriers

Whilst many noted positive experiences, some pharmacy users described negative experiences where pharmacy staff seemed to lack awareness about how to communicate effectively, and empathetically, with people with hearing loss. In some cases, pharmacists used inappropriate strategies, for example:

Oh, they've tried to speak a bit more clearly. The thing is when somebody tries to do that they tend to [excessively] slow down their speech [PU13].

I think if staff had a bit more information about how difficult it can be and the experiences of people with hearing loss… how to deal with people with different levels of hearing loss… people think that you're either profoundly deaf and you're signing, or you're okay. And if you've got a hearing aid then you're okay, but… your hearing aid's still picking up the background noise [PU07].

Some pharmacy users used strategies to cope with communication difficulties, such as pretending to hear, which they recognised could create issues when communicating about medicines. Such maladaptive strategies were described by pharmacy users who attempted to hide, or expressed feeling embarrassed about having or disclosing, hearing loss:

I just pretend I've heard because it's easier. It's a bit more difficult somewhere like pharmacy because they may be asking you something that's really important… now I know the questions… what's your name, what's your address, I'm not saying, sorry, what did you say? [PU08].

Use of hearing loops, which transmit auditory information to hearing aids, was omitted from most pharmacy users accounts. However, some divulged that they were not aware of their pharmacy having a hearing loop, or whether they were actively used, switched on, or reflect a ‘box-ticking’ exercise [PU13]. Pharmacists reported variable implementation and use of hearing loops, noting barriers related to cost and end-users lacking awareness of how to use them.

Pharmacists reported limited awareness of strategies, tools, and guidance to support communication with people with hearing loss, noting that the AIS^15^ provides limited advice on implementation. Instead, pharmacists relied on their own experiences and “common sense” [CP06] about how to communicate effectively with this population, identifying a training gap:

The accessible information standards… didn't come with a massive training programme of how you should implement them, they didn't come with any training in fact… we do need some training [CP01].

### 5.2 Facilitators

Further to the behavioural strategies pharmacy users adopted to cope with environmental barriers, some suggested that communicating clearly, and addressing barriers to communication, was a joint responsibility between them and pharmacy staff:

As much as I'm expecting them [the pharmacist] to make themselves loud and clear, I've got to make myself concentrate more because of the situation we're in, it's a two-party thing [PU13].

Some pharmacy users described examples of positive strategies which pharmacists used to facilitate clear communication about medications they collected, whereby they are “very very clear” [PU14] when verbally checking the person's understanding of how and when to take medicines.

Pharmacists discussed a range of positive strategies they used to identify people with hearing loss, which they viewed as necessary to facilitate effective communication. Without individual's disclosing their hearing loss, pharmacists relied on other cues to recognise people with hearing loss, including speech differences, people giving unrelated answers to questions, asking staff to repeat, and watching their lips closely. Strategies pharmacists perceived to facilitate communication ranged from maintaining eye contact, “rolling out the words quite clearly and slowly” to facilitate lip-reading [CP04], highlighting important information in medication leaflets, providing extra notes on medicine labels, pointing to information, and using teach back: having the person repeat instructions or demonstrate use of a new medication or device:

As a pharmacist it's like recognising when they've got hearing loss, moving them to a quiet space, making sure that the light is on your face so that they can lipread easily, making sure you're facing them, maintaining eye contact, not chewing, not wearing a mask - which is challenging over the last few years… Repeating things, clarifying things. Talking at a measured pace, not too fast, not too slow. Not shouting; shouting doesn't help as it just distorts the sound [CP07].

Both pharmacists and pharmacy users suggested potential solutions to improve communication in the pharmacy, summarised in Table 3. Based on the barriers experienced, some pharmacy users suggested that pharmacy staff would benefit from further training on communicating accessibly and empathetically with people with hearing loss, to recognise that some people may be self-conscious about having hearing loss:

The training [should] be, if somebody says sorry, what did you say, you realise that they don't hear well, so you speak much better… more enunciated, maybe louder, not necessarily… a video saying this is how difficult it is for me and this is what would help… to bring it home to them what a difference they could make to somebody who is nervous, edgy about their hearing loss [PU08].

**Table 3:** Suggested solutions and targets for future interventions to facilitate effective communication.

Target	Interventional strategies	Indicative pharmacy user quotes	Indicative pharmacist quotes
Organisation	• Access to patient records with information about sensory needs • Develop an electronic flag for hearing loss in patient records	‘Information sharing from the GP about somebody that's got some kind of a hearing loss’ [PU08]	‘It's more about identifying and flagging… so that the information comes to us’ [CP01]
Environment	• Clear signage in the pharmacy that sensory needs are recognised • Discreet method to disclose sensory needs and disabilities at the counter • Design ways to maintain privacy and confidentiality with people who may have difficulty hearing and may not disclose these difficulties	‘I don't see any behavioural things or visual things… to say we are aware of or, can we help you in the following circumstances’ [PU09] ‘If they did have something like on your prescription… So that the onus isn't always on me’ [PU07] ‘Anybody could overhear what I was saying to the chemist, any member of the public… there's no confidentiality’ [PU15]	‘Emphasise that we are here to help… in our practice leaflet’ [CP04] *‘*A card is probably the most discreet way, you can just pass it to the counter assistant’ [CP02] ‘… running the risk there of breaking a bit of confidentiality… shouting someone's address… to make sure they can hear’ [CP08]
Pharmacy staff	• Improve pharmacists' awareness of strategies, adaptations, and assistive technologies	‘If staff had a bit more information about how difficult it can be and the experiences of people with hearing loss’ [PU07]	‘If you could just say it into your iPhone and print it off… I'm guessing that technology exists… we don't know about it’ [CP01]
People with hearing loss	• Encourage hearing aid use • Overcome stigma and encourage disclosure of hearing problems	‘once you accept… that you wear a hearing aid, you have no problem. But I have quite a few friends who have hearing aids but refuse to wear them because they think it makes them look old’ [PU05]	‘access to - not to put it back on the patient - to hearing aids’ [CP07] ‘that patient group, who have a greater need, aren't always as forthcoming to say they need the help… sometimes they're too embarrassed’ [CP02]

Pharmacists reiterated the need for clear guidance and support around communication strategies. They suggested digital interventions to facilitate communication, including speech-to-text apps or assistive listening devices. Such technology did not appear to be available to pharmacists currently, and they cited barriers to engagement, especially older people's digital literacy and exclusion:

If you could just say [information] into your iPhone and print it off, then that would be handy. So, from our perspective, ways of doing that would be useful and I'm guessing that technology exists… we don't use it, because we don't know about it [CP01].

Pharmacists suggested GPs should share information about a person's hearing loss with community pharmacy teams and proposed the development of an electronic flagging system, based at the GP, to automatically alert the pharmacist about a person's sensory needs when their record is accessed:

A software which flags it straight to the pharmacy team… that would be the ideal, because then that takes it away from everyone, it's clear to the pharmacy team, so the patient doesn't need to worry about identifying themselves [CP02].

## 6 Discussion

This study reports the barriers and facilitators to effective communication according to pharmacists and pharmacy users who self-identified as having hearing loss and were mostly White British, situated in North-West England. Both groups identified distinct barriers and facilitators to communication and saw a need for solutions. Overall, pharmacy users perceived that staff did not always know how to manage situations, whilst most pharmacists felt they knew what to do but were unsure or unable to implement them in current contexts.

### 6.1 Context of community pharmacy

Community pharmacies in the UK are commissioned through a national contract with the NHS: the Community Pharmacy Contractual Framework.^22^ Community pharmacy services have expanded to include multiple and varied non-dispensing services, helpfully summarised by Rutter and Barnes (2024),^23^ as part of an effort to improve access to primary care. Many commentators argue that NHS funding provided by central government is inadequate for meeting the ever-growing demand for services,^24^ with funding reductions and workforce pressures affecting community pharmacy.^25^ It is within this context that pharmacists in this study discussed workload and time constraints as barriers to communication with people with hearing loss, and pharmacy users expressed having limited opportunities to communicate about matters beyond collecting prescriptions.

Pharmacists recognised that time constraints and competing workloads led to problematic short cuts, such as condensing information about medications for people with hearing loss, thus increasing the risk of adverse medication events.^13^^,^^26^ This concern has been noted by others.^11^ Time constraints also hindered implementation of adaptations, such as moving to a private consultation room to facilitate spoken communication. Ferguson and Shan (2016) point out that providing written information for people with hearing loss, an adaptation suggested by pharmacists in this study, takes time, which could exacerbate these time pressures.^17^ It is vital to identify solutions to excessive workloads and time constraints to ensure all pharmacy users receive complete information about their medications. As community pharmacy settings vary considerably in their layout, size, organisation, barriers to communication varied by location. Both groups reported high levels of noise as communication barriers, mirroring others' findings.^13^ Yet, pharmacy users highlighted supermarket-based pharmacies as particularly noisy environments, indicating a need for more hearing-friendly environments.

Whilst pharmacy users were aware that community pharmacy offered more services than prescription filling, most used the pharmacy exclusively for this purpose. This aligns with evidence that older people are more likely to seek health advice from GPs than pharmacists^27^ and that people with hearing loss are more likely to report medication side effects to GPs than pharmacists.^12^ The extent to which this is because communication is easier in one-on-one GP appointments than a noisy pharmacy setting, as identified here, is unknown.

Pharmacy users reported that their interactions with staff were typically limited to providing personal details when collecting prescriptions, and many ordered prescriptions online. In line with other studies,^13^^,^^26^ pharmacists raised concerns that people may not be managing their medications appropriately, and that they could not monitor medication adherence and side effects. It perhaps indicates a need to promote interactions so that pharmacists can fulfil their role of overseeing medication use and adherence.

### 6.2 Implications for disclosing hearing loss

Pharmacy users recognised they must adapt their behaviour to facilitate successful communication. Yet, they tended not to disclose their hearing loss in pharmacies. This related to feelings of embarrassment about having hearing loss, as others have reported,^10^, ^11^, ^12^, ^13^^,^^26^ which seemed to relate to gender and generational expectations. This connects with findings on negative stereotyping which associates hearing loss and hearing aids with ageing and disability.^28^ Some pharmacy users said they did not disclose their hearing loss partly because they considered it unnecessary as they had little interaction with pharmacy staff. However, pharmacists viewed not disclosing hearing loss as a major barrier to address. As others argue,^13^ when hearing loss goes undisclosed, patients likely do not receive the support they need to communicate, as hearing loss is not externally visible. Whilst disclosure of sensory impairment could improve pharmaceutical care,^7^ pharmacists often rely on spotting cues of hearing loss (lipreading, hearing aids, misunderstandings).^13^ Once identified, they can offer adaptations when time and knowledge permits. It is important to take seriously and overcome perceived negative consequences of disclosure, such as embarrassment connected with stereotypes.^13^^,^^29^ Pharmacists and pharmacy users suggested clear signage and symbols indicating that support is available could promote recognition, and encourage disclosure of, communication need.

### 6.3 Implications for implementing communication strategies

Pharmacy users and pharmacists reported privacy concerns when pharmacists raised their voice to communicate, which others have noted as a barrier to service use.^27^^,^^30^^,^^31^ Unlike other research,^10^ the pharmacists in this study displayed knowledge about communication strategies that help people with hearing loss, and an awareness of their responsibility to adhere to the AIS. Nonetheless, pharmacy users thought pharmacists could be more empathetic regarding their hearing loss, and pharmacists stated that implementation of adaptations was lacking due to time constraints, lack of guidance, and system barriers, including limited access to patient records. It would seem that training, organisational changes and formal guidance are needed to improve this. The Scottish Sensory Impairment and Pharmaceutical Care Study developed a free online training resource for healthcare professionals, carers, friends and family of older people with sensory loss,^11^^,^^13^^,^^26^ which meets the training needs identified here.^32^ This course is optional, thus limiting uptake. It would be beneficial for such education to be embedded in pharmacy undergraduate training programmes.

It is well-established that face coverings, Perspex screens at counters, and social distancing during the COVID-19 pandemic exacerbated communication barriers for people with hearing loss.^33^ This was reflected in the present study. Although these measures are no longer a requirement in UK healthcare settings, many are still practiced today and are recommended by the World Health Organisation.^34^ Healthcare professionals should bear this in mind when wearing a face covering or communicating from behind a Perspex screen.

There is a need to develop tailored interventions to support access and address communication barriers, which may involve assistive technology. Research has started scoping the views of hard-of-hearing people on a mobile health app to facilitate communication with pharmacists, who welcomed the proposition whilst identifying considerations about privacy and data security.^35^ A scoping review of assistive technologies to support the medication management of people with hearing and/or visual impairment found that only four out of 17 technologies were available to the public, and two of those were unavailable in the UK.^36^ However, there are now a range of accurate speech-to-text apps, some of which are freely available.^37^

### 6.4 Strengths and limitations

As data were collected online in the aftermath of the COVID-19 pandemic, participants' perspectives were likely affected by recent restrictions and experiences. As all data were collected online, or by telephone, the findings may not reflect older people who are less digitally engaged or excluded.^38^ The fact that many pharmacy users had a university degree could explain the widespread use of online services and why engagement with community pharmacy was reportedly unimportant. While this might not be the case for less digitally-engaged or less advantaged groups, recent findings indicate that many older people order prescriptions online.^38^ Readers should take into account contextual factors in terms of pharmacy user's self-disclosed hearing loss, primarily White-British ethnicity and situatedness in North-West England, along with the reflexive positionalities of the study team, when considering the transferability of the findings.

## 7 Conclusion

This study identified environmental barriers contributing to missed opportunities for effective communication with people with hearing loss in community pharmacy, relating to time pressures, noise, COVID-19 protective measures (face coverings), and limited confidentiality, which must be addressed. Differing views on the need to have regular contact with community pharmacy, and pharmacy users' personal feelings about having hearing loss, related to pharmacy users reported and perceived tendency not to disclose hearing loss. Implementing visible signage to recognise sensory needs could enable pharmacy users to disclose their hearing loss and communication needs, to enable pharmacists to provide the necessary support. To achieve this, there is a need to improve pharmacists' ability to implement strategies, adaptations, and potentially digital interventions to facilitate communication with people with hearing loss, and to evaluate the effectiveness and acceptability of these interventions, to ensure equitable access to services for all pharmacy users.

## CRediT authorship contribution statement

**Shanice Thomas:** Writing – review & editing, Writing – original draft, Visualization, Validation, Software, Resources, Project administration, Methodology, Investigation, Formal analysis, Data curation, Conceptualization. **Jane Griffiths:** Writing – review & editing, Methodology, Investigation, Funding acquisition, Formal analysis, Data curation, Conceptualization. **Gabrielle Saunders:** Writing – review & editing, Visualization, Validation, Resources, Project administration, Methodology, Investigation, Funding acquisition, Formal analysis, Data curation, Conceptualization. **Denham Phipps:** Writing – review & editing, Visualization, Validation, Resources, Project administration, Methodology, Investigation, Funding acquisition, Formal analysis, Data curation, Conceptualization. **Chris Todd:** Writing – review & editing, Visualization, Validation, Methodology, Investigation, Funding acquisition, Formal analysis, Conceptualization. **Penny Lewis:** Writing – review & editing, Visualization, Validation, Supervision, Resources, Project administration, Methodology, Investigation, Funding acquisition, Formal analysis, Data curation, Conceptualization.

## Funding sources

This work was supported by the 10.13039/501100000770University of Manchester Interdisciplinary Research Institute: Interdisciplinary Research Recovery Fund 2021–22**.** GS was supported by the National Institute for Health and Care Research (NIHR) Manchester Biomedical Research Centre, CT and PL by NIHR Applied Research Collaboration-Greater Manchester, and PL by NIHR Greater Manchester Patient Safety Research Collaboration. The views expressed are those of the authors and not necessarily those of the NHS, the NIHR, the Department of Health and Social Care, or its partner organisations.

## Declaration of competing interest

The authors declare that they have no known competing financial interests or personal relationships that could have appeared to influence the work reported in this paper.

## Appendix A Supplementary data

Interview and focus group topic guides Image 1
