# Supplementary material for: Exploring the barriers and facilitators to effective communication with people with age-related hearing loss in community pharmacy settings
Source: Explor Res Clin Soc Pharm. 2025 May 13;19:100573. doi: 10.1016/j.rcsop.2025.100573 (PMC12142350; doi:10.1016/j.rcsop.2025.100573)
Supplement: Supplementary file 1 — Interview and focus group topic guides [file mmc1.docx]

**“If somebody doesn’t hear what you say… change the way you say it”: A qualitative study into effective communication with people with age-related hearing loss in community pharmacy settings.**

**Supplementary Data**

Appendix 1: Summary of interview topic guide for pharmacy user participants

1. What is a community pharmacy to you?

- Role/responsibility of the pharmacy team.
- Expectations of services and professionals.

1. Which type of community pharmacy do you use? (retail/high street/independent/GP)

- How often do you use this pharmacy?
- Do you use any other pharmacy?

1. What do you use a community pharmacy for?

- What ways do you communicate with the pharmacy (face-to-face, email, telephone)?

1. Tell me about your experiences when trying to access or use a service at a community pharmacy.

- Could you tell me about your most recent negative/positive experience?
- How would you describe your relationship with the pharmacy staff?
- Can you describe any challenges you have faced?
- What do you think the pharmacy staff can or should do to help you communicate?

1. Do you think your community pharmacy is aware of your hearing loss?

- How did your pharmacist find out?
- Do you know if your hearing loss is mentioned in your patient record? If so, how would you feel if this information was shared with the pharmacy?

1. Have you ever been in a situation where the pharmacy staff have not recognised you have hearing loss and you have not mentioned it?

- Have pharmacy staff ever asked you about your communication support needs?

1. Do you think your pharmacist knowing about your hearing loss causes a change in the service you receive?
2. Has the pandemic affected your experiences when using pharmacy services? If so, how?

- Can you tell me about any challenges you faced in the pharmacy environment, after Covid-19 rules were introduced?

1. How would you describe your relationship with the pharmacy staff after the introduction of Covid-19 rules?
2. How do you feel your experiences when using community pharmacy services can be improved?

Appendix 2: Summary of focus group and interview topic guide for community pharmacist participants

1. What do you think are the current problems that people with age-related hearing loss face in a community pharmacy setting?

- Are there any services that older people with hearing loss have difficulty using?
- What do you think it is like to have hearing loss?

1. What have you noticed when interacting with someone with hearing loss?

- How do you know if they have understood what you have said to them?
- Situational probes: new medications or polypharmacy; over the counter consultations and knowing patients’ needs.
- Do other pharmacy staff find it difficult to interact with people with hearing loss?
- How do you know that someone has hearing loss?
- Do you have a way of recording patients who have hearing loss and their needs? Is any information about a patient’s hearing loss ever shared with you by other healthcare services?

1. Is there anything the patient does or could do that makes it easier/harder when interacting with them? Is there anything that you do that makes it easier/harder?
2. How has the Covid-19 pandemic impacted your interactions with people with hearing loss?

- Probes: use of masks and clear screens; experiences with remote contact (e.g., by phone)
- With limits on the number of people that can enter the pharmacy, were there times where only the patient was able to go in without a caregiver? How did that affect the consultation?

1. Is there anything you could think of to improve patient experience?

- Probes: Technology to aid communication (hearing loops, assistive listening devices); moving to the consultation room; written resources; delivery services; pharmacist skills and knowledge?
